# Hospital Meetings, &C.

**Published:** 1898-07-23

**Authors:** 


### HOSPITAL MEETINGS, &C.

### GREAT NORTHERN CENTRAL HOSPITAL.

#### Opening of Another Ward.

One of the circular wards of the Great Northern Central Hospital, which for want of funds has remained unoccupied since it was built a year or two ago, was opened for immediate use on July 19th, 1898. The Bishop of Islington conducted a hearty service in the ward, at which a choir of nurses and Miss Adela Bona provided the music. The Bishop then gave a short address and formally declared the ward open. In the first place he expressed the general regret at the recent death of Mr. C. T. Murdock, M.P., who had been connected with the hospital for thirty years and chairman during the latter part of the time. He had greatly looked forward to being present at the present ceremony. The Bishop went on to say that opening the ward might be regarded as a venture of faith on the part of the committee, for which the district owed much gratitude. The hospital, he understood, had been removed from Caledonian Road because the accommodation there had been inadequate for the requirements of the neighbourhood. In the ward they were opening there were twenty-five beds, which meant that 400 more patients per annum could be received. No one could feel a deeper interest in the work than the local clergy, who saw the benefit the sick derived from a sojourn in a well-ordered hospital such as the one of which he was speaking. Not only were the patients restored physically, but the moral influence exercised by the kind Christian care they received was spiritually healing and beneficial. Most charitable enterprises were ventures of faith, and were justified by results. It remained for the inhabitants of the district to justify the action of the committee and find funds for maintaining it. Mr. Sawbridge, chairman of the house committee, proposed a vote of thanks to his lordship. He sketched the rise and growth of the hospital, and said that the present buildings had cost £80,000 to erect, and the maintenance had cost a further £60,000. He dwelt upon the importance of a regular income, pointing out that of the large number of householders only about 400 subscribed annually to the funds. The new ward would cost £1,000 per annum to maintain. The Diamond Jubilee Committee had handed over £800 (a subsequent speaker corrected this; the sum was £900) towards the maintenance for the first year. Nor should the efforts of the Ladies' Committee be overlooked; it had collected £16,000 for various purposes. When the question of income was satisfactorily provided for the hospital would rank as one of the best in London. The vote was seconded and carried unanimously, and a numerous company re-assembled in the board-room, where tea, coffee, and light refreshments were provided. The ward is completely furnished, and looked very fresh and pretty. The beds were made, and the patients will be moved in at once.

